# The Beneficial Effects of UM206 on Wound Healing After Myocardial Infarction in Mice Are Lost in Follow-Up Experiments

**DOI:** 10.3389/fcvm.2019.00118

**Published:** 2019-09-18

**Authors:** Evangelos P. Daskalopoulos, Kevin C. M. Hermans, Jacques Debets, Agnieszka Strzelecka, Peter Leenders, Lily Vervoort-Peters, Ben J. A. Janssen, W. Matthijs Blankesteijn

**Affiliations:** Department of Pharmacology and Toxicology, Cardiovascular Research Institute Maastricht (CARIM), Maastricht University (UM), Maastricht, Netherlands

**Keywords:** myocardial infarction, cardiac remodeling, heart failure, Wnt/frizzled signaling, receptor blockade, peptide fragment

## Abstract

**Introduction:** An inadequate wound healing following myocardial infarction (MI) is one of the main etiologies of heart failure (HF) development. Interventions aiming at improving this process may contribute to preserving cardiac function after MI. Our group, as well as others, have demonstrated the crucial role of Wnt/frizzled signaling in post-MI remodeling. In this overview, we provide the results of different studies aimed at confirming an initial study from our group, in which we observed beneficial effects of administration of a peptide fragment of Wnt5a, UM206, on infarct healing in a mouse MI model.

**Methods:** Mice were subjected to permanent left coronary artery ligation, and treated with saline (control) or UM206, administered via osmotic minipumps. Cardiac function was assessed by echocardiography and hemodynamic measurements, while infarct size and myofibroblast content were characterized by (immuno)histochemistry.

**Results:** In total, we performed seven follow-up studies, but we were unable to reproduce the beneficial effects of UM206 on infarct healing in most of them. Variations in dose and timing of UM206 administration, its manufacturer and the genetic background of the mice could not restore the phenotype. An in-depth analysis of the datasets revealed that the absence of effect of UM206 coincided with a lack of adverse cardiac remodeling and HF development in all experimental groups, irrespective of the treatment.

**Discussion:** Irreproducibility of experimental observations is a major issue in biomedical sciences. It can arise from a relatively low number of experimental observations in the original study, a faulty hypothesis or a variation in the experimental model that cannot be controlled. In this case, the lack of adverse cardiac remodeling and lung weight increases in the follow-up studies point out to altered experimental conditions as the most likely explanation.

## Introduction

Heart failure (HF) is a devastating condition that frequently results from myocardial infarction (MI). The development of HF is generally thought to be the consequence of adverse cardiac remodeling (1). Although the initial remodeling of the infarct area after MI is generally considered to be beneficial, excessive remodeling leads to dilatation of the entire left ventricle, inducing a deleterious effect on cardiac function (2). This has resulted in the concept that interventions targeting post-MI wound healing can be cardioprotective.

In the past decades, extensive efforts have been made to study the post-MI wound healing process, in order to discover novel therapeutic targets. Several pathways have been identified and tested in animal models (2). Our lab has a longstanding interest in the role of Wnt signaling in cardiac remodeling following MI and the potential of this pathway for interventions aiming to improve wound healing. Following the initial description of the activation of Wnt signaling post-MI (3), several studies from our group and others have provided evidence for the beneficial effects of Wnt-signaling-related interventions on the wound healing post-MI (4). Our group has published data on an intervention with a peptide fragment of Wnt5a– named UM206– in a mouse MI model (5). UM206 was designed to interfere with the interaction between Wnt and its receptors, members of the frizzled protein family. In this study, subcutaneous administration of UM206 (*n* = 26) or saline (*n* = 17)–via an osmotic minipump–resulted in a reduction of the infarct size by almost 40% and a >3-fold increase in myofibroblast content in the infarct area, when compared to saline treatment. The end-diastolic volume of the LV more than doubled in saline-treated mice at 5 weeks post-MI, but only increased ~35% in the UM206-treated animals. The ejection fraction (EF) was 31 ± 3% in the UM206-treated group, compared to 17 ± 3% in saline-treated animals. Furthermore, the increase in lung weight, a sign of fluid retention in the lung due to compromised pump function, was significantly reduced and the mortality, which amounted 35% in the saline-treated group at 5 weeks post-MI, was completely prevented in the UM206-treated group. These observations indicated that HF development was attenuated in this relatively large group of UM206-treated animals (5).

Following this successful study, we decided to test variations in the dosage regimen and timing and thus gain more insight into the cellular target(s) of UM206 and the phase(s) of the wound healing where the compound displays the optimal activity, in order to shed light onto its exact mechanism(s) of action. We initially observed that interventions with UM206 starting at 2 weeks post-MI were almost as successful as administration of UM206 for 5 weeks following MI. However, after two positive studies the beneficial effect of UM206 appeared to be lost. In this manuscript, we provide an overview of the available experimental data regarding the variable effects of UM206 on infarct healing and discuss the possible interpretations of our findings.

## Methods

### Overview of the Studies

In this overview, we present the results from seven different studies in which we assessed the effects of UM206 on infarct healing. UM206 was administered subcutaneously via an osmotic minipump at a dose of 6 μg/kg/day, with the exception of study G where a higher dose (150 μg/kg/day) was used. In all studies except for study D, Swiss mice were used as they show a strong tendency to develop left ventricular (LV) dilatation and HF after MI (6). In all studies, the administration of UM206 started either directly or after a specified period following the induction of MI. The dosage regimens are graphically represented in Table 1, where the orange bars indicate the UM206-treatment periods and the gray bars indicate the saline-treatment periods.

**Table 1:**
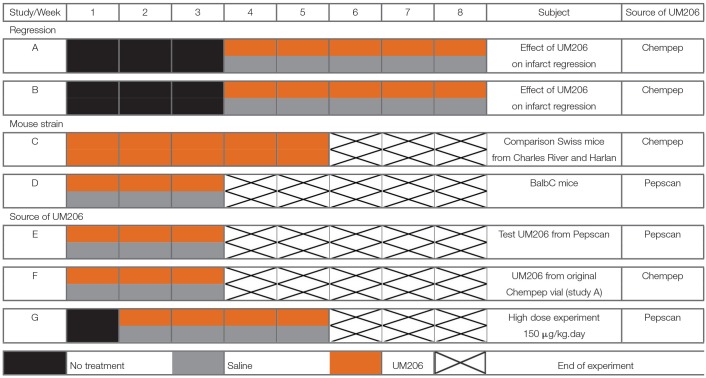
Schematic overview of the included studies, illustrating the treatment protocols with either UM206 (orange bars) or saline (gray bars).

*Periods where no treatment was administered are represented by black bars. In total, data from seven different studies were included in this overview. The length of the studies, the specific subject of each study and the source of UM206 are indicated in the respective columns*.

In studies A and B, infarct healing was followed for 8 weeks with UM206 administered for the final 5 weeks (first 3 weeks were treatment-free). In study C, Swiss mice from two different suppliers (Charles River and Harlan) were subjected to a 5 weeks UM206 treatment (starting shortly after MI induction). In study D, the influence of the genetic background was explored by using BALB/c rather than Swiss mice. In study E, the oxidized (circular) form of UM206 was administered whereas in all other studies the linear (reduced) form was applied. Because UM206 obtained from different sources was used in the previous studies (see below), the UM206 from the batch used in the original study as well as in study A was used again in study F. Finally, in study G, UM206 was administered at a maximally soluble dose of 150 μg/kg/day.

### Origin and Administration of UM206

UM206 was produced either by ChemPep Inc., Miami FL, USA or by Pepscan Therapeutics, Lelystad, The Netherlands. UM206 is a 13 amino acid peptide derived from an area of high homology between multiple Wnt molecules. It has a molecular weight of 1,427 Da and the following sequence: Ac-CNKTSEGMDGCEL-NH_2_. In previous experiments, this peptide was found to inhibit the frizzled-1 and−2 mediated canonical Wnt signaling induced by Wnt3a *in vitro* (5). The UM206 compound was administrated subcutaneously via osmotic minipumps (Alzet 2006; Durect, Cupertino CA, USA).

### Animal Surgery

An overview of the executed studies can be found in Table 1. Male Swiss mice were used (10–12 weeks of age at the start of the study) in all studies with the exception of study D, where male BALB/c mice were used. The animals were supplied by Charles River, Leiden, The Netherlands, but in study C, Swiss mice from Charles River and Harlan (Horst, The Netherlands) were compared to each other. Animals had free access to food and water. MI was induced under isoflurane gas anesthesia (2–3%) using a stereomicroscope (Leica MZ FL III, Leica Switzerland) as previously described (6). Briefly, animals were placed on a heating pad in supine position, endotracheal intubation was performed under direct laryngoscopy and mechanical ventilation was maintained with a small animal respirator (Hugo Sachs, MiniVent, tidal volume: 180–250 μl, rate: 200–250 breaths/min, depending on the strain). After thoracotomy, the lateral branch of the left anterior descending (LAD) coronary artery was ligated with a 6-0 prolene suture, just proximally to the main bifurcation. Successful ligation was verified by visual inspection of the LV apex for myocardial blanching, indicating interruption of the coronary blood flow. The chest cavity and skin were closed in layers with 5-0 polysorb sutures. Animals were gradually weaned from the respirator. All experimental procedures were approved by the Committee for Animal Research of Maastricht University and were performed by highly trained and experienced users that were blinded to the treatment groups.

### Echocardiography

Left ventricular (LV) dimensions and function were assessed under isoflurane anesthesia (2–3% for induction and 1–2% for maintenance) as previously described (5). B-mode echocardiographic recordings were made in midpapillary short-axis and parasternal long-axis using a Vevo 2100 imaging platform (VisualSonics, Toronto, ON, Canada) with a 30 MHz transducer. Data were derived from images in end diastole and peak systole and average values over at least three different cycles were used. From the long-axis images, LV area (LVA) as well as the length of the LV lumen from base to apex (LVL) were determined. The end-diastolic volume (EDV) and end-systolic volume (ESV) were calculated from the area and length measurements as (8 × LVA^2^)/(3π × LVL) in diastole and systole, respectively, and ejection fraction (EF) was deducted. All measurements and analysis were performed by experienced users that were blinded to the treatment groups.

### Hemodynamics

Hemodynamic measurements were performed as previously described (7). Briefly, mice underwent urethane anesthesia (2.5 mg/kg i.p.), then a high-fidelity catheter tip micromanometer (Mikro-tip 1.4F; SPR-671, Millar Instruments, Houston TX, USA) was inserted through the right carotid artery into the LV cavity. The heart was then stimulated by an i.v. ramp infusion of dobutamine (Sigma Aldrich, Saint Louis, MO, USA) using a microinjection pump (model 200 Series, KdScientific, Boston, MA, USA). Every 2 min the infusion rate of dobutamine was increased by 2.5 ng/g body weight^−1^ min^−1^ up to 20 ng/g body weight^−1^ min^−1^ and contractility data were recorded.

### Histological Analysis

Histological analysis of the infarcted hearts was performed as previously described (6). Briefly, mouse hearts were longitudinally cut in half, perpendicular to the septum, fixed in 4% paraformaldehyde solution for 24 h and embedded in paraffin. Sections (4 μm) were cut and stained with the AZAN technique. Infarct size (percentage of the LV length) was measured using a computerized morphometry system (Qwin, Leica). Alpha smooth muscle actin (αSMA) monoclonal antibody (Sigma, dilution 1:1,000) was used to identify myofibroblasts in the infarct area. The relative myofibroblast area was determined using the Qwin morphometry system with manual exclusion of vascular smooth muscle cells and expressed as myofibroblast area/total tissue area (%).

### Statistical Analysis

Data are expressed as means ± SEM. For comparisons between individual groups, Student's *t*-test was used. A *P* < 0.05 (two-sided) was considered to be statistically significant. Comparisons of multiple groups were performed using one-way ANOVA with Dunnett's *post-hoc* test. Again, *P* < 0.05 (two-sided) were considered to be statistically significant. All statistical analyses were performed using GraphPad software (version 7).

## Results

### Effects of UM206 Treatment on Mortality After MI

One of the most remarkable effects of the UM206 treatment in our previously published study was the complete prevention of mortality post-MI (5). In Swiss mice, mortality due to HF is typically around 30%, 5 weeks after induction of MI (6), a figure that was confirmed in the saline-treated group in our original study. An overview of the mortality rates of the mice in all seven studies is provided in Table 2. It is remarkable to note that in all studies the mortality was found to be considerably lower than in the previously published study, irrespective of whether the animals received UM206 or saline treatment.

**Table 2:** Overview of mouse mortality in all follow-up studies.

**Study**	**% saline (dead/total)**	**% treated (dead/total)**	**Mortality before start of treatment**	**Total mouse number**	**Total mortality**
**Regression**					
A	0% (0/6)	0% (0/9)	11	26	11
B	0% (0/6)	14% (2/14)	2	22	4
**Mouse strain**					
C	N/A	5% (1/20)	N/A	20	1
D	15% (2/13)	0% (0/13)	N/A	26	2
**Source**					
E	0% (0/10)	13% (1/8)	N/A	18	1
F	0% (0/7)	0% (0/4)	N/A	11	0
G	0% (0/6)	0% (0/8)	3	14	3

*N/A, “not available” (no deaths recorded)*.

### Effects of Late Administration of UM206 (Studies A and B)

**Figure 1:**
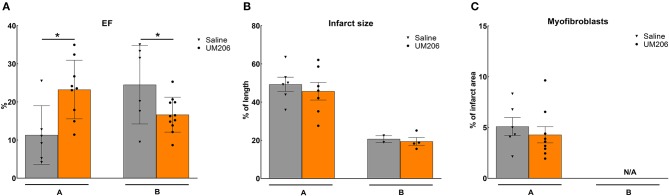
Regression studies (Studies A and B): Cardiac function, infarct size, and myofibroblast counts. **(A)** Cardiac function (as measured by ejection fraction [EF%]), **(B)** Infarct size determination (measured by IHC and expressed as % of tissue length), and **(C)** Myofibroblast count (measured by IHC and expressed as % of infarct area). EF, ejection fraction; IHC, immunohistochemistry; N/A, not available. Data are presented as mean ± SEM. *P < 0.05.

These experiments were designed to study the effects of UM206 on the later phases of infarct healing to explore whether this intervention could induce regression of the infarct size. To this end, the administration of UM206 started at 3 weeks after infarction and continued until 8 weeks post-MI. As shown in Figure 1, in the initial experiment (study A) an improved EF was observed (Figure 1A), which was accompanied by a significant reduction in EDV and ESV (Table 3). In study A, UM206 treatment also resulted in a significant reduction in lung weight. However, these findings were not paralleled by a smaller infarct size (Figure 1B), an increase in myofibroblast numbers (Figure 1C) or improved hemodynamics (Table 3), as observed in our original study (5). Moreover, a second experiment with an identical experimental setup (Study B) showed a significant decline in EF (Figure 1A) and no significant effects on any of the other parameters tested (Figure 1B and Table 3). Unfortunately, due to a technical failure in the tissue processing we could not determine the myofibroblast content in study B.

**Table 3:** Regression studies (Studies A and B): echocardiographic parameters (EDV and ESV), hemodynamic characteristics determination (dP/dt max and dP/dt min), and heart and lung weight measurements.

	**Study A**		**Study B**	
	**Saline**	**UM206**	**Saline**	**UM206**
EDV (cm^3^)	0.278 ± 0.024	0.195 ± 0.014^***^	0.192 ± 0.021	0.204 ± 0.012
ESV (cm^3^)	0.250 ± 0.027	0.151 ± 0.015^***^	0.149 ± 0.023	0.171 ± 0.012
dP/dt max (mmHg/sec)^†^	3345 ± 1210	5088 ± 942	5495 ± 620	3797 ± 326^*^
dP/dt min (mmHg/sec)^†^	−2020 ± 500	−2292 ± 472	−2839 ± 353	−2330 ± 149
HW/BW	0.0051 ± 0.0002	0.0047 ± 0.0002	0.0047 ± 0.0003	0.0047 ± 0.0001
LW/BW	0.0072 ± 0.0011	0.0050 ± 0.0001^*^	0.0050 ± 0.0003	0.0060 ± 0.0005

*The dP/dt max^†^ and dP/dt min^†^ data represent the effect of dobutamine on the dP/dt corrected for the baseline (dP/dt max or min value minus the dP/dt baseline value respectively). EDV, end diastolic volume; ESV, end systolic volume; HW/BW, heart weight to body weight ration; LW/BW, lung weight to body weight ratio. Data are presented as mean ± SEM*.

* *P < 0.05*,

*** *P < 0.001*.

### Effect of Mouse Strain and Supplier (Studies C and D)

**Figure 2:**
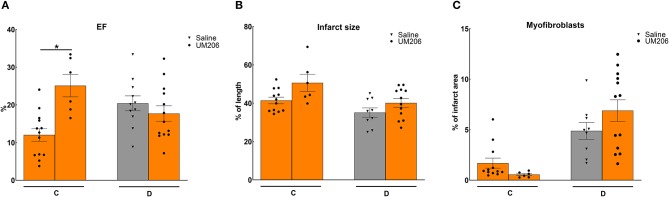
Mouse strain studies (Studies C and D): Cardiac function, infarct size, and myofibroblast counts. **(A)** Cardiac function (as measured by ejection fraction [EF%]), **(B)** Infarct size determination (measured by IHC and expressed as % of tissue length), and **(C)** Myofibroblast count (measured by IHC and expressed as % of infarct area). EF, ejection fraction; IHC, immunohistochemistry. Data are presented as mean ± SEM. *P < 0.05.

In previous studies, we observed profound effects of mouse strain and supplier on infarct healing (6). Therefore, we compared Swiss mice from two different suppliers (Charles River and Harlan, Study C) and studied the effects of UM206 on infarct healing in BalbC mice (study D). The EF was found to be significantly higher in the UM206-treated Swiss mice supplied by Harlan (Figure 2A), but this was not accompanied by a significant difference in infarct size (Figure 2B), myofibroblast content (Figure 2C) or any of the other parameters listed in Table 4. Along similar lines, no beneficial effects of UM206 administration were observed in BalbC mice for any of the tested parameters (Figure 2 and Table 4).

**Table 4:** Mouse strain studies (Studies C and D): echocardiographic parameters (EDV and ESV), hemodynamic characteristics determination (dP/dt max and dP/dt min), and heart and lung weight measurements.

	**Study C**		**Study D**	
	**Swiss Harlan**	**Swiss C.-R**	**BalbC**	**BalbC**
	**UM206**	**UM206**	**Saline**	**UM206**
EDV (cm^3^)	0.228 ± 0.012	0.210 ± 0.017	0.123 ± 0.009	0.130 ± 0.006
ESV (cm^3^)	0.202 ± 0.013	0.159 ± 0.018	0.099 ± 0.008	0.108 ± 0.007
dP/dt max (mmHg/sec)^†^	2999 ± 499	4291 ± 861	5017 ± 876	4293 ± 656
dP/dt min (mmHg/sec)^†^	−1774 ± 310	−2666 ± 775	−2646 ± 436	−2464 ± 330
HW/BW	0.0042 ± 0.0001	0.0046 ± 0.0001	0.0062 ± 0.0004	0.0065 ± 0.0005
LW/BW	0.0081 ± 0.0007	0.0058 ± 0.0005	0.0100 ± 0.0019	0.0106 ± 0.0017

*The dP/dt max^†^ and dP/dt min^†^ data represent the effect of dobutamine on the dP/dt corrected for the baseline (dP/dt max or min value minus the dP/dt baseline value respectively). EDV, end diastolic volume; ESV, end systolic volume; HW/BW, heart weight to body weight ration; LW/BW, lung weight to body weight ratio. Data are presented as mean ± SEM*.

### Effect of Oxidative State, Source, and Dose of UM206 (Studies E–G)

**Figure 3:**
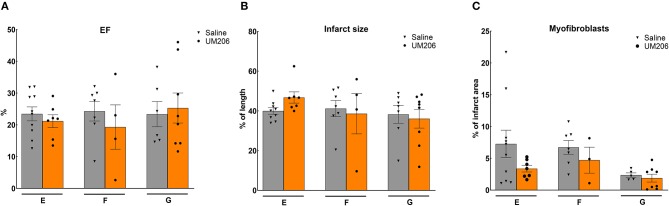
Different source/dose of UM206 studies (Studies E, F, and G): **(A)** Cardiac function (as measured by ejection fraction [EF%]), **(B)** Infarct size determination (measured by IHC and expressed as % of tissue length), and **(C)** Myofibroblast count (measured by IHC and expressed as % of infarct area). EF, ejection fraction; IHC, immunohistochemistry. Data are presented as mean ± SEM.

To rule out any effects of the oxidative state, source and dose of UM206, we performed three additional experiments; the results are presented in Figure 3 and Table 5. UM206 contains two Cys residues which, when oxidized, can form a disulfide bond that circularizes the peptide. In study E, we compared the administration of this circular form of UM206 with saline treatment and did not observe any effect of the administration on the parameters tested. In study F, we administered UM206 from the original badge produced by Chempep, identical to the compound used in our original study (5). Again, no effect of this therapy was observed on any of the parameters tested. Finally, we decided to increase the dose of UM206 from 6 μg/kg/day, as used in all previous experiments, to 150 μg/kg/day (Study G). This dose was achieved by inserting a saturated solution of UM206 in the minipumps. Unfortunately, similarly to the previous experiments we did not observe any beneficial effects of this high-dose administration of UM206 when compared to saline administration.

**Table 5:** Studies on different sources and dosing of UM206: echocardiographic parameters (EDV and ESV), hemodynamic characteristics determination (dP/dt max and dP/dt min), and heart and lung weight measurements.

	**Study E**		**Study F**		**Study G**	
	**Pepscan 6** **μg/kg/day**		**Chempep 6** **μg/kg/day**		**Pepscan 150** **μg/kg/day**	
	**Saline**	**UM206**	**Saline**	**UM206**	**Saline**	**UM206**
EDV (cm^3^)	0.187 ± 0.011	0.182 ± 0.007	0.142 ± 0.008	0.137 ± 0.020	0.194 ± 0.020	0.190 ± 0.019
ESV (cm^3^)	0.145 ± 0.012	0.144 ± 0.008	0.109 ± 0.010	0.115 ± 0.026	0.152 ± 0.021	0.148 ± 0.022
dP/dt max (mmHg/sec)^†^	3915 ± 587	3349 ± 622	2201 ± 432	4039 ± 1711	3150 ± 561	3826 ± 446
dP/dt min (mmHg/sec)^†^	−1941 ± 302	−1841 ± 335	−924 ± 232	−1861 ± 559	−1537 ± 356	−1767 ± 247
HW/BW	0.0044 ± 0.0001	0.0043 ± 0.0001	0.0051 ± 0.0004	0.0053 ± 0.0005	0.0048 ± 0.0003	0.0045 ± 0.0001
LW/BW	0.0057 ± 0.0005	0.0049 ± 0.0002	0.0070 ± 0.0019	0.0080 ± 0.0022	0.0065 ± 0.0011	0.0071 ± 0.0011

*The dP/dt max^†^ and dP/dt min^†^ data represent the effect of dobutamine on the dP/dt corrected for the baseline (dP/dt max or min value minus the dP/dt baseline value respectively). EDV, end diastolic volume; ESV, end systolic volume; HW/BW, heart weight to body weight ration; LW/BW, lung weight to body weight ratio. Data are presented as mean ± SEM*.

## Discussion

In this manuscript, we provide an overview of seven studies, performed in our laboratory, on the effects of UM206 on the wound healing after MI. These studies were performed to confirm the strong protective effect of this treatment on adverse cardiac remodeling, as previously illustrated by less dilatation of the left ventricle, improved cardiac function and lower lung weights, as well as improved survival (5). This beneficial effect of UM206 treatment on infarct healing could partially be confirmed in the first follow-up study, although the increase in myofibroblast numbers could not be reproduced. In the other studies, no significant differences have been observed in the infarct healing UM206-treated groups compared to saline-treated controls.

Irreproducibility of experimental data is a major issue in biomedical sciences. Researchers from the pharmaceutical companies Bayer and Amgen have systematically investigated this issue and found that the results of 75–91% of published experimental studies could not be reproduced in their own laboratories (8, 9). This illustrates that we are dealing with a considerable number of variables that are difficult to control, but that are of paramount importance for the outcome of an experiment. It is quite evident that when one is confronted with a discrepancy between subsequent studies, a scrutinous comparison of the experimental conditions is executed to identify potential differences. First, the surgical team that performed the operations is highly experienced and has not changed over the years so this is an unlikely factor. Originally, the Swiss mice were obtained from Charles River, but in order to exclude a possible effect of the genetic background we also tested Swiss mice from Harlan as well as BALB/c mice, with similar results. This excludes the animal background and surgery as a plausible explanation for the variations observed in the effects of UM206.

Another variable that we addressed was the source of UM206. In most of the studies, we made use of UM206 produced by ChemPep (Miami FL, USA) but we also tried UM206 produced by Pepscan (Lelystad, the Netherlands). No consistent differences in effects were observed between the peptides produced by these two companies, which is in complete agreement with the identical analytical data sheets provided with the compounds.

Since, UM206 is a peptide of 13 amino acids with two Cys residues located at positions 1 and 11, the peptide can form an internal disulfide bond when it is placed under oxidative conditions. Alternatively, dimeric or even multimeric complexes can also be–theoretically–formed, although this is obviously more likely to occur when the compound is dissolved at a high concentration. Given the low concentration of UM206 in the minipumps (4 μmol/l), the formation of multimers is unlikely. *In vitro* incubation of UM206 in osmotic minipumps under oxidative conditions revealed that virtually all of the UM206 will have formed disulfide bonds–mostly the internal variant–within 24 h (personal communication Dr. Nicolas Dailly and Dr. Peter Timmerman, Pepscan Lelystad). In previous *in vitro* studies, we have found the oxidized form of UM206 to be inactive as an antagonist, so this could explain the lack of effect observed in the follow-up studies. However, then the question remains unanswered how the compound could be administered in the active form in the initial studies, since the mode of administration (osmotic minipump) was the same.

A third issue is the position of the UM206 peptide in the Wnt/frizzled complex. The crystal structure of the *X*Wnt8/frizzled-8 cysteine-rich domain was published (10), showing that it is unlikely that the loop of the Wnt protein where UM206 is derived from is in direct physical contact with the frizzled protein. This finding obscures the pharmacological mechanism of action of this peptide. In the meantime, several researchers have provided evidence that Wnt and frizzled interact in a complex with other proteins, including the co-receptor LRP5/6 (11), and it is quite likely that UM206 interferes somehow with this complex formation. We cannot exclude that variations in the components involved in the formation of this complex determine the effectiveness of UM206, but it is unclear how this could have changed over time.

A remarkable observation in the current set of experimental data is that the lung weights in the saline-treated animals in the original study were considerably higher than the lung weights in the follow-up studies, despite similar infarct lengths. This suggests that overt HF induced by MI was only present in the first studies, but the development of the cardiac remodeling response was less severe in the latter ones. It is obvious that a therapy aiming at preventing adverse cardiac remodeling is ineffective when this adverse remodeling does not take place. This raises the question whether there have been any changes in the experimental conditions between the initial and follow-up studies that could explain these deviating outcomes. At this point, we can only speculate about experimental conditions, such as e.g., housing conditions, which could influence the infarct healing to the extent that heart failure development after MI was prevented. An alternative explanation could be that changes in the gut microbiome, which is increasingly recognized as factor regulating the wound healing after MI (12), have affected the heart failure development after MI over time.

A plausible explanation for irreproducible results can be that the underlying hypothesis is wrong and the positive results, observed in the initial experiment, are actually the result of chance. In this case, this explanation for the lack of reproducibility does not seem to hold. First of all, in the initial study, a relatively large number of animals (saline: *n* = 17, UM206-treated: *n* = 26) was included (5), supporting the robustness of the statistical analysis of these data. Moreover, we tested UM206 in a swine model of ischemia/reperfusion and one of the most prominent findings of this study was a reduced infarct size, similar to what we observed in our initial mouse study (13). In this context, it is relevant to note that several other authors have demonstrated beneficial effects on infarct healing of pharmacological inhibition of Wnt signaling (14). This includes interventions at the level of Wnt secretion (15–17), the β-catenin destruction complex (18), and the β-catenin transcription complex (19, 20). Repression of the methylation of the Wnt inhibitory factor 1 gene was shown to improve the differentiation of cardiac progenitor cells into cardiomyocytes, resulting in preserved LV wall thickening and improved LV functional parameters (21). In all of these studies, the inhibition of Wnt signaling induced a reduction in infarct size, similar to what we observed in our initial UM206 study. Furthermore, Foxy-5, a peptide derived from the same region of Wnt5a as UM206, has been shown to reduce metastatic spread of prostate cancer cells, underscoring the pharmacological potency of Wnt5a fragments (22). Taken together, explaining the inconsistent results from our mouse studies by an incorrect underlying hypothesis is becoming increasingly unlikely.

## Conclusions

The current overview of multiple studies on the effect of UM206 on infarct healing in mice suggests that changes in the experimental model had a profound effect on the results. In this case, the lack of HF development in the follow-up studies most likely has obscured the beneficial effects of the compound on wound healing post-MI. In the meantime, studies by several other groups have confirmed the beneficial effects of pharmacological Wnt inhibition on infarct healing by targeting the pathway at different levels. This supports the validity of the concept that inhibition of Wnt signaling after MI can be a promising novel approach to improve wound healing and preserve cardiac function.

## Data Availability

The raw data supporting the conclusions of this manuscript will be made available by the authors, without undue reservation, to any qualified researcher.

## Author Contributions

ED and KH are the shared first authors and responsible for literature search, conduction of experiments, data analysis and interpretation, as well as manuscript preparation, and editing. JD, AS, PL, and LV-P helped with data collection. BJ helped with data analysis and manuscript editing. WB is the senior author and was responsible for study conception, overseeing data collection and analysis, literature search, as well as manuscript preparation, and editing.

### Conflict of Interest Statement

The authors declare that the research was conducted in the absence of any commercial or financial relationships that could be construed as a potential conflict of interest.
